# 

**Published:** 1917-04-14

**Authors:** 


### CONTENTS.

| | PAGE |
|---|---|
| **Medical Research in India** ... ... ... ... ... | 19 |
| **The Easter Bank Holiday, 1917** ... ... ... | 20 |
| **Hospital and Institutional News** ... ... ... | 21 |
| What is Truth?—An Important Post Unfilled—Proposed Large Gift of Land to Newcastle Royal Infirmary—Developments at Warrington Infirmary—The Sanitation of Osborne—Research into Referred Pain—Making the Wounded a Bone of Contention—Another Hospital for Shell-Shock Cases—Reaping the Tares—An Uphill Fight in Kensington—German Women of the Red Cross—The Woman House Surgeon after the War—The Campaign against Venereal Diseases—Ratepayers Only May Compete at Derby—Bradford Guardians on Hospital Architecture—Foresight at the Retford Hospital—Curious Proposal by Australian Saturday Fund—The Rationing Problem in Hospitals—Memorials—This Week's Drug Market. | |
| **Recent Work on Artificial Pneumothorax** ... | 25 |
| Benefits and their Limitations. | |
| **The Surgery of Military Spinal Injuries** ... | 26 |
| Some Indications for its Employment. | |
| **The Uniting of the Nations** ... ... ... ... | 27 |
| Will Britons in the Homeland Awake and Act? By Sir Henry Burdett, K.C.B., K.C.V.O. | |
| **Parliament and the Profession** ... ... ... | 29 |
| **Hospital Architecture and Construction** ... | 30 |
| Leeds General Infirmary Extensions. | |
| **National Insurance Act Developments** ... ... | 32 |
| The Necessity for Amending Legislation. | |
| **From Across the Seas** ... ... ... ... | 34 |
| The Position of Margarine in the Food World—News from New Zealand—Hospital Workers' Trade Union. | |
| **The Editor's Letter-Box** ... ... ... ... | 34 |
| Summer Knitting. | |
| **The Matrons' and Sisters' Department** ... | 35 |
| The Profession of British Nurses: Plain Words on Registration.—III. | |
| Round the Hospitals. | |
| The Mountain Bears!—The Lady, British Nurses, Noodles and Know-Nothings—British Includes Irish—How Not to Marry—New Zealand's Practical Interest in Nurses. | |
| **Food in Wartime** ... ... ... ... ... ... | 37 |
| Guardians and Economy. | |
| **The Patriotism of the Nurse-Training Schools** | 38 |
| XIII. Some Lancashire Institutions. | |
| **Matrons' and Sisters' Appointments** ... ... | iv |
| **Passing Events and Topics...** ... ... ... ... | iv |
| **Hospital and Institutional Results** ... ... | iv |
| **The Institutional Worker** (Suppl.) | 1 |
| Outfit for Factory Medicine Cupboard—Answers to March Question. | |
| Regulating and Checking Stationery Supplies. | |
| Question for April. | |

**By Sir HENRY BURDETT, K.C.B., K.C.V.O.**

